# The first case report of distal 16p12.1p11.2 trisomy and proximal 16p11.2 tetrasomy inherited from both parents

**DOI:** 10.3325/cmj.2023.64.339

**Published:** 2023-10

**Authors:** Leona Morožin Pohovski, Ivona Sansović, Katarina Vulin, Ljubica Odak

**Affiliations:** Department of Medical and Laboratory Genetics, Endocrinology and Diabetology, Children’s Hospital Zagreb, University of Zagreb, School of Medicine, Zagreb, Croatia

Recurrent copy number variants in the chromosomal region 16p11.2 are among the most frequent genetic causes of neurodevelopmental disorders. The increasing prevalence of brain structural anomalies is also associated with 16p11.2 deletions and duplications. We report on a four-year-old boy with microcephaly, trigonocephaly, and dysmorphic features. The patient also exhibited motor delay and autism spectrum disorder. Microarray analysis showed a single-copy gain of a 1.187 kb segment in the 16p12.1p11.2 region and a two-copy gain of a 525 kb segment in the 16p11.2 region. Parental analysis revealed a 1.7 Mb duplication at the 16p12.1p11.2 (BP1-BP5 region) in the father and a 525 kb duplication in the 16p11.2 region (BP4-BP5) in the mother. The patient inherited the entire abnormality from each parent and, as a result, presented with partial trisomy of the 16p12.1p11.2 region and partial tetrasomy of the 16p11.2 region. The MLPA P343 Autism-1 Probemix was used to verify the copy number gains in the 16p11.2 region detected by chromosomal microarray analysis. Double duplications are very rare chromosomal rearrangements. The phenotype for distal 16p12.1p11.2 trisomy (BP1-BP3) and proximal 16p11.2 (BP4-BP5) tetrasomy is unknown. To our knowledge, this is the first patient described in the literature who inherited 16p11.2 duplications from both parents.

Recurrent copy number variants in the chromosomal region 16p11.2 are among the most frequent genetic causes of neurodevelopmental disorders. The increasing prevalence of brain structural anomalies is also associated with 16p11.2 deletions and duplications. A triplication of the 16p11.2 region is quite rare; therefore, the associated phenotype is unknown. We present the first case of “double duplication” within the 16p11.2 genomic region (BP4-BP5).

## Case report and cytogenomic studies

**Figure 1:**
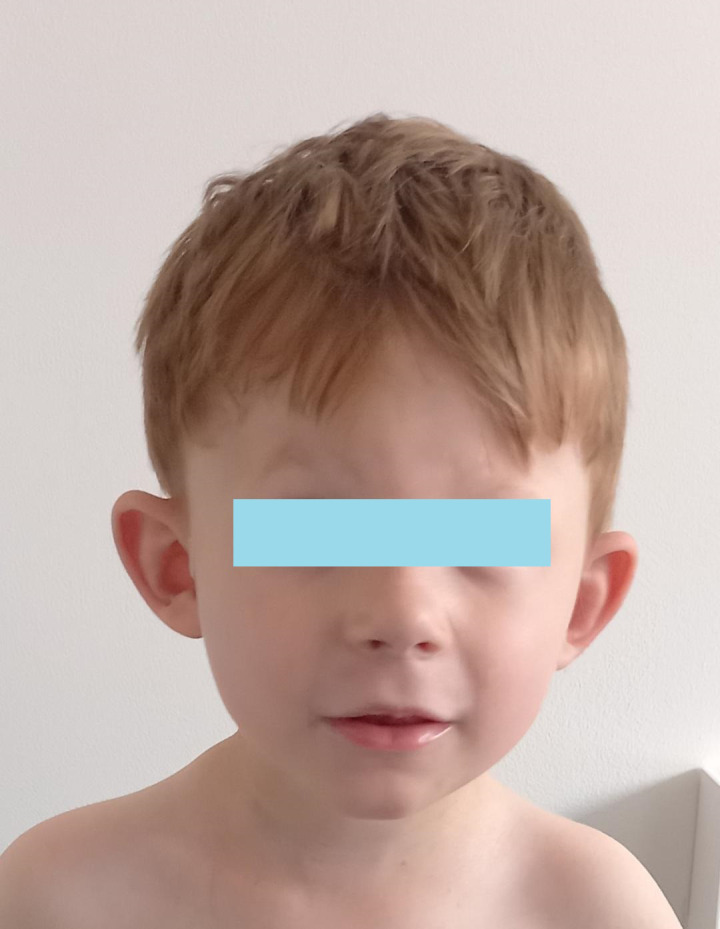
The patient at the age of four years.

**Figure 2:**
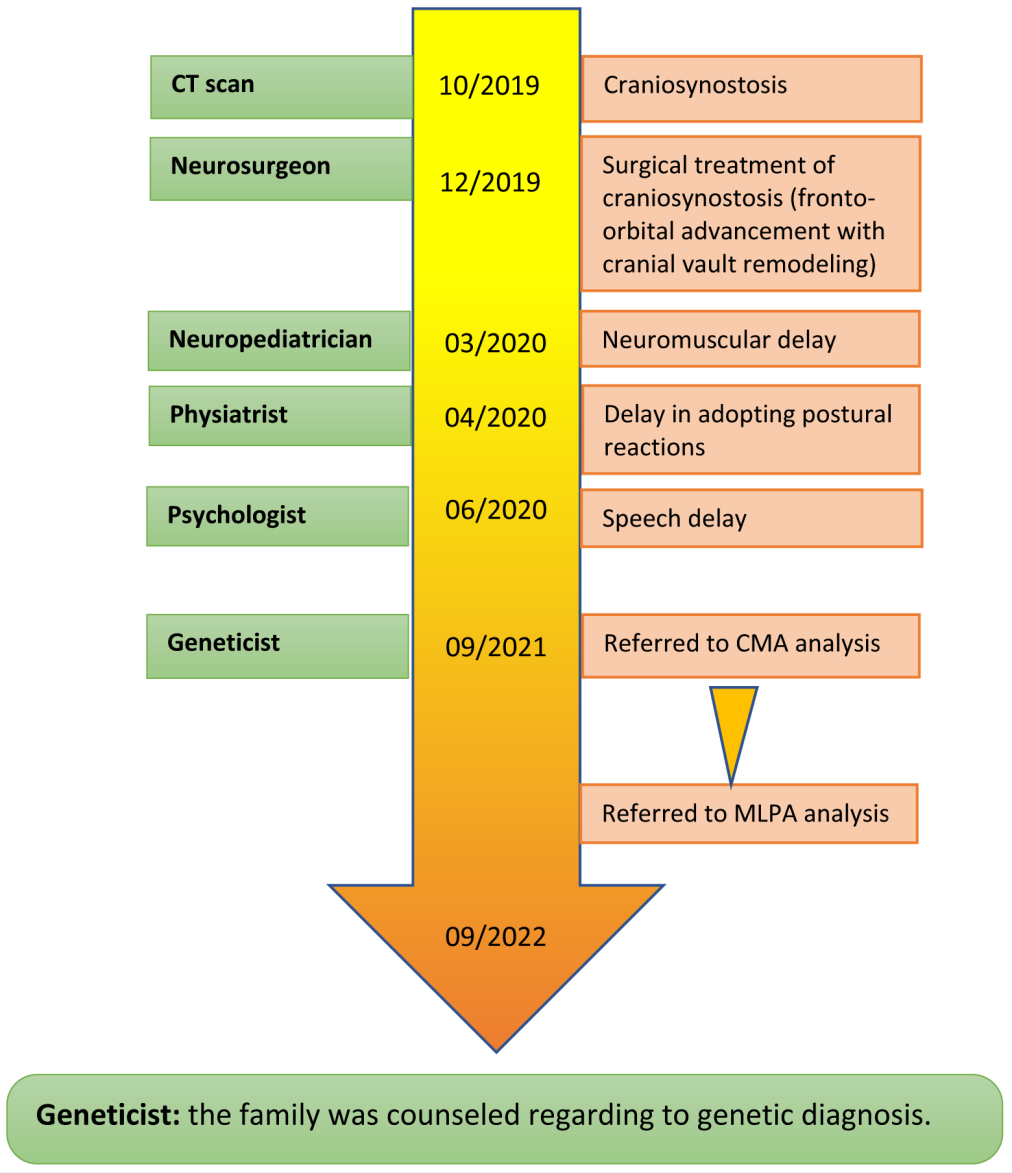
A timeline of events and diagnostic procedures. CMA - chromosomal microarray analysis; MLPA - multiplex ligation-dependent probe amplification.

**Figure 3:**
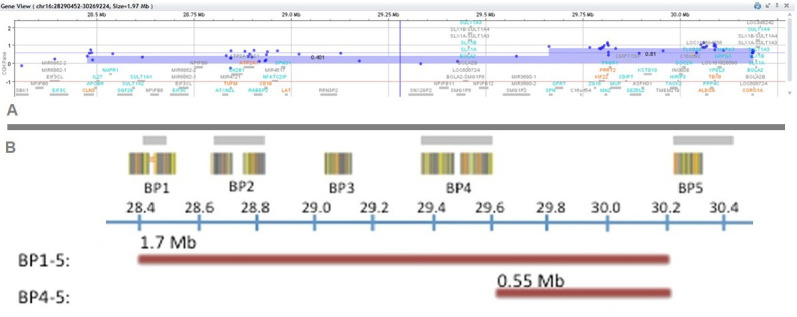
(**A**) Genome view of array showing a single-copy gain and two-copy gain in the 16p11.2 region. (**B**) Red lines represent BP1-5 duplication inherited from the father (1.7 Mb) and BP3-5 duplication inherited from the mother (0.525 Mb).

We report on a four-year-old boy with a unique partial trisomy of the 16p12.1p11.2 region and a partial tetrasomy of the 16p11.2 region identified by chromosomal microarray. The patient presented with microcephaly, trigonocephaly, dysmorphic features (including a round face, prominent ears, and epicanthal folds), motor delay, and autism spectrum disorder (Figure 1). The timeline of the most important events and diagnostic procedures is shown in Figure 2. Chromosomal microarray was carried out with a whole-genome Agilent 8x60K kit (Human Genome CGH Microarray, Agilent Technologies, Santa Clara, CA, USA). The analysis with Agilent CytoGenomics software (V5.1.2.1) identified a single-copy gain of a 1.187 kb segment in the 16p12.1p11.2 region and a two-copy gain of a 525 kb segment in the 16p11.2 region. An array analysis revealed a common chromosome 16p11.2 duplication in the mother, spanning approximately 525 kb (GRCh38 chr16: 29662635_30188030), and a 1.7 Mb duplication in the 16p12.1p11.2 region in the father (GRCh38 chr16: 28475372-30188030). The patient inherited the entire abnormality from each parent, and consequently presented with a partial trisomy of the 16p12.1p11.2 region and a partial tetrasomy of the 16p11.2 region (Figure 3). The results of the arrays were confirmed by a multiplex ligation-dependent probe amplification analysis with SALSA Probemix P343-C3 (MRC Holland, Amsterdam, the Netherlands), which contains 11 probes for the 16p11.2 region.

## Discussion

We present the first case of “double duplication” within the 16p11.2 genomic region (BP4-BP5). Double duplications are very rare chromosomal rearrangements, with unknown clinical manifestations. Ballif et al reported on a case involving a complex *de novo* abnormality that encompassed both a duplication in the 16p12.2p12.1 and a triplication region in the 16p12.1p11.2 region (1). The distal duplication spanned 1.1 Mb, while the triplication spanned 5.7 Mb, flanked by duplications. Wallace et al described a *de novo* 16p11.2 triplication, spanning approximately 597 kbp, while the proband’s mother had a *de novo* duplication in the same region (2). The triplication in the proband was presumed to have originated via a nonallelic homologous recombination during maternal meiosis. In contrast to these cases, our patient exhibited a complex chromosomal abnormality characterized by a distal 16p12.1p11.2 trisomy (BP1-BP3) and a proximal 16p11.2 (BP4-BP5) tetrasomy, but as a result of inherited duplications from both parents. The clinical presentations of the described patients are outlined in Table 1. Recent findings suggest that reciprocal copy number variations within the 16p11.2 region can result in multiple phenotypes (3). Specifically, individuals with deletions often present with macrocephaly and obesity, whereas individuals with duplications present with microcephaly and underweight. These contrasting phenotypes support the hypothesis of dosage-sensitive genes residing within the 16p11.2 region. This hypothesis aligns with gene expression analyses, which consistently demonstrate a robust correlation between the expression levels of genes within the affected fragment and genomic copy number, implying a limited argument of dosage compensation (4). Nevertheless, it remains uncertain whether an increased dosage of the 16p11.2 locus alone leads to a discernibly distinct clinical phenotype, given the presence of duplications in the patient’s healthy parents. One plausible explanation is that this duplication acts as a potential risk factor, necessitating a “second hit” to manifest as a clinically significant phenotype. This “second hit” may involve an additional copy of the same locus, under the assumption that one or more of the implicated genes are sensitive to changes in dosage. An alternative explanation for our patient's clinical presentation, particularly his autistic features, could be the presence of additional mutations within the genes associated with autism spectrum disorder (ASD). Alternatively, an increased copy gain at 16p11.2 might contribute to the susceptibility to ASD. From the genomic perspective, this case illustrates a recessive disorder induced by copy number gains, as the patient’s parents, who harbored three copies, exhibited no evident abnormalities, while the patient, carrying four copies, presented with a distinctive clinical phenotype. However, the precise mechanisms underlying these observations warrant further investigation.

**Table 1:** Genotype-phenotype correlation of published cases with triplication of the 16p11.2 region

	Patient 5 in the study by Ballif et al (1)	Patient in the study by Wallace et al (2)	Our patient
Imbalance	dup(16)(p12.2p12.1) dn trp(16)(p12.1p11.2) dn	trp(16)(p11.2)	(16)(p12.1p11.2)x3 pat, (16)(p11.2)x4 mat pat
Sex	F	M	M
Age at evaluation	10y 11m	2y 7m	4y 2m
Size of imbalance	1.1 Mb duplication; 5.7 Mb triplication	597 kb triplication	1.2 Mb x3; 525 kb x4
Craniofacial	head circumference -1 SD; round face with full cheeks	head circumference N.A.; broad forehead	head circumference -3 SD; trigonocephaly; sparse medial eyebrow; full cheeks
Mouth & Jaw	high-arched palate; retrognathia; wide mouth; long philtrum; thin upper lip	N.A.	wide mouth; flattened long philtrum; thin upper lip
Eyes	narrow and slightly short palpebral fissures; relative hypertelorism; ptosis (right eye); strabismus (left eye); hyperopia	mild hypertelorism; bilateral epicanthal folds; mild visual impairment	bilateral epicanthal folds; narrow and slightly short palpebral fissures
Ears	low-set and posteriorly rotated ears; hyperacusis	mildly protuberant ears with thickened superior helices	low-set prominent ears
Nose	short nose with wide nasal bridge; round nasal tip; anteverted nares	broad nasal root	wide nasal bridge
Cardiovascular	persistent tachycardia	right-sided aortic arch and two small, nonhemodynamically significant arterial septal defects	normal heart US
Skeletal Muscular	height -3 to -4SD; weight -2SD; hypotonia; bridged palmar creases (left hand); short fifth fingers; prominent finger-tip pads; bilateral hallux valgus, minimal 2-3 toe syndactyly	wide spaced nipples, hypotonia, lordosis	heigh -1 SD; weight -0.6 SD; hypotonia; bilateral clinodactyly of the fifth finger; prominent finger tip pads
Gastrointestinal and nutrition	feeding difficulties in infancy	excessive drooling, chronic constipation	picky eater
Psychomotor and cognitive delay	significant delay, IQ of 42 (WISC-IV at age 9)	suspected mild intellectual disability, developmental coordination disorder	borderline range (WPPSI-IV)
Behavioral	friendly and talkative; ADHD; anxiety and nervousness leading to nail biting and skin picking to the point of trauma	autism spectrum disorder	autism spectrum disorder symptoms; earlier frequent episodes of meltdowns; hyperkinetic disorder
Other	growth hormone deficiency	allergies	retractile testicles

*Abbreviations: NA - not available; SD - standard deviation; ADHD - attention deficit hyperactivity disorder; WISC-IV - Wechsler Intelligence Scale for Children – Fourth Edition; WPPSI-IV - Wechsler Preschool and Primary Scale of Intelligence, Fourth Edition.

In conclusion, we extensively described the observed phenotype in a male patient with partial duplication and a partial double duplication within the 16p11.2 chromosome region. By establishing connections between our patient and two previously reported cases, which represent the sole instances of triplication of the 16p11.2 region in the literature, we elucidated the repercussions of increased copy numbers at the 16p11.2 locus. The duplications identified in our patient's parents suggest that the 16p11.2 duplication alone may not be pathogenic but could necessitate additional rare and/or common alternations, or potentially detrimental epigenetic factors, to manifest as a clinical phenotype. However, a tetrasomy, whether through double duplication or expansion of a parental duplication in this region, may culminate in a phenotype affecting cortical, skeletal, and muscular development, facial appearance, as well as psychomotor and cognitive capabilities.

Further studies, including the identification and thorough phenotypic characterization of additional cases with tetrasomy, are needed to complement ongoing endeavors and address fundamental questions related to one of the most prevalent genetic contributors to neurodevelopmental abnormalities.
